# Emotional and behavioral alterations and their relationship with executive functioning in the pediatric population with autism spectrum disorder and epilepsy: a comparative study

**DOI:** 10.3389/fpsyg.2023.1219177

**Published:** 2023-06-30

**Authors:** Alejandro Cano-Villagrasa, Francisco J. Moya-Faz, Antonio Cortés-Ramos, Miguel López-Zamora

**Affiliations:** ^1^Health Sciences PhD Program, Departamento de Psicología Básica, Facultad de Ciencias de la Salud, Universidad Católica de Murcia UCAM, Murcia, Spain; ^2^Universidad Internacional de Valencia, Valencia, Spain; ^3^Cátedra Psicogeriatría, Facultad de Medicina, Universidad Católica San Antonio de Murcia, Murcia, Spain; ^4^Departamento de Psicología Evolutiva y de la Educación, Facultad de Psicología y Logopedia, Universidad de Málaga, Málaga, Spain

**Keywords:** ASD, epilepsy, childhood, behavior, emotion

## Abstract

**Introduction:**

The pediatric population with Autism Spectrum Disorder (ASD) and epilepsy presents behavioral and emotional alterations that hinder their correct developmental maturation as well as their integration in different contexts such as school, family or social. This population shows atypical behavioral and emotional patterns, with difficulties in emotional regulation, understanding of emotions, aggressiveness, or low frustration tolerance. They also present alterations in executive functions, which significantly influence the emotional and behavioral characteristics of this population. Research suggests that epilepsy worsens the emotional, behavioral, and executive functioning status.

**Objective:**

To explore differences in behavioral, emotional, and executive functioning profile in individuals with a diagnosis of ASD, epilepsy, and ASD with epilepsy.

**Method:**

In this quasi-experimental and cross-sectional study, a total of 170 participants were selected and distributed into three groups: a group of participants with ASD, a group with epilepsy, and a group of participants with ASD and epilepsy. The SENA, BASC-3, and ENFEN tests were administered to verify the behavioral, emotional, and executive functioning profile in the three groups.

**Results:**

The results indicate that individuals diagnosed with ASD and epilepsy present greater emotional, behavioral, and executive functioning alterations compared to those who only present ASD or epilepsy.

**Conclusion:**

Individuals with ASD and epilepsy present significant alterations in emotional, behavioral, and executive functioning processes, which hinder their adaptation to different contexts, as well as decreasing their quality of life and that of their family.

## Introduction

Autism Spectrum Disorder (ASD) and epilepsy are two disorders that fall under the category of Neurodevelopmental Disorders (American Psychiatric Association, 2013). On one hand, individuals diagnosed with ASD are characterized by limitations in reciprocal social interaction, communication, and restricted interests that result in repetitive or stereotyped behaviors (Sevilla et al., 2014). On the other hand, those who have epilepsy, in addition to the seizures associated with this disorder, show alterations in linguistic, motor, or sensory competences (Vania and De La Barra, 2013). People with either of these two disorders, in addition to the specific symptomatology of their pathology, often present difficulties in cognitive functioning, learning, attention, and sensory processing (Angulo et al., 2020), negatively affecting the correct developmental maturation and the quality of life of families and individuals diagnosed with them (García-Peñas, 2009). However, the situation is worsened if both disorders are present in the same person. Thus, if a person with ASD also has comorbid epilepsy, this will lead to an increase in the frequency and severity of autism symptoms, causing greater difficulties in areas such as communication, social interaction, learning, behavior, and emotional regulation (Gutiérrez-Ruiz, 2019; Cano-Villagrasa et al., 2023).

Epilepsy and ASD are neurological disorders that affect the brain; however, a comprehensive understanding of their connection and differentiation using brain signals has yet to be fully elucidated. The scholarly article authored by Wadhera (2021) brings to the forefront the development of an automated diagnostic model based on electroencephalography (EEG) with the intention of unraveling the association between epilepsy and ASD. The fundamental objective of this investigation is to acquire a deeper comprehension of the intricate relationship between these two neurological conditions and ascertain their detectability through EEG analysis techniques. The proposed model makes effective use of EEG data acquired from patients diagnosed with epilepsy, patients diagnosed with ASD, and a control group devoid of either condition, ultimately facilitating the construction of an automated classification model. The discernible outcomes of this study reveal marked distinctions in the distinctive characteristics of the brain network between the epilepsy and ASD cohorts. The automated diagnostic model successfully achieves noteworthy accuracy in categorizing patients with epilepsy and those with ASD, as compared to the control group. These consequential findings postulate the existence of a relationship between epilepsy and ASD at the level of brain connectivity, which can be discerned through the utilization of automated EEG analysis techniques. The proposed model harbors the potential to substantially enhance the accuracy of diagnosis and further our understanding of these neurological conditions, thereby engendering noteworthy implications for treatment modalities and early intervention strategies.

In a related vein, the work conducted by Wadhera and Mahmud (2022) delves into the exploration of brain networks in the context of ASD and epilepsy, while simultaneously investigating their interconnectedness. Employing an innovative approach that amalgamates machine learning methodologies with functional magnetic resonance imaging (fMRI) and EEG data, this study endeavors to attain insights into the intricate network patterns within the brains of individuals afflicted by ASD, epilepsy, and control subjects. Machine learning algorithms elucidate compelling disparities in the salient characteristics of brain networks amongst the ASD, epilepsy, and control groups, effectively unveiling unique and distinctive connectivity patterns intrinsic to each group. Furthermore, this research reveals an inherent relationship between ASD and epilepsy, as evidenced by shared characteristics observed in the brain networks. The utilization of machine learning techniques in this investigatory endeavor yields a powerful toolset, facilitating the comprehensive analysis and comprehension of intricate brain networks implicated in both ASD and epilepsy. This pioneering approach exhibits the potential to significantly enhance the precision of diagnostic processes associated with these neurological conditions, while concurrently augmenting our comprehension of their intricate interplay and interdependencies.

The main characteristics of a person with comorbid ASD and epilepsy include impairments in social interaction and communication skills, as well as the presence of restricted and repetitive interests and behaviors (Málaga et al., 2019). However, individuals with this comorbidity often experience additional physical and mental health conditions, as well as behavior problems that can affect their lives and families, such as aggression, lack of control, emotional dysregulation, among others. Among these, one that stands out for its manifestations at all levels of these patients is the presence of emotional and behavioral problems (Dominick et al., 2007; Hurtig et al., 2009), which include aggression, tantrums, or self-injurious behaviors. Sleep, feeding, and sensory disorders are also common, as well as intellectual disability or learning difficulties, in addition to mental health conditions such as attention deficit hyperactivity disorder, anxiety, obsessive–compulsive disorder, and tic disorders (Dominick et al., 2007).

Regarding the etiology of this comorbidity, authors such as López Rodríguez and Cañadas Pérez (2018) and Ruggieri (2020) suggest the presence of shared underlying mechanisms that may play a role in the existence of anxiety or depression disorders in the child population with ASD and epilepsy. On the one hand, there are social factors that trigger a barrier in the interaction of the person with ASD and epilepsy, leading to communicative limitations with other individuals. This will lead to inefficient emotional management, with a diminished self-perception of the correct execution of social strategies, generating a situation of discomfort and frustration that is related to a state of high anxiety. On the other hand, there will be biological factors that will play a fundamental role in the onset of anxiety or depression in the person with ASD and epilepsy. In these patients, there is identified a hyperactivation of the amygdala, as well as other structures of the central nervous system such as the hippocampus and the brainstem, structures that end up triggering alarm responses in the person with ASD and epilepsy when detecting a threat in social contexts.

In this sense, it is vital to consider the possibility that the child population with the joint comorbidity of ASD and epilepsy shows depressive episodes and an acute state of hyperactivation compared to those children who only have ASD or epilepsy (Vania and De La Barra, 2013). People with ASD and epilepsy are four times more likely to develop depression than the general population, being considered one of the most common mental health conditions among them (Benítez Gort et al., 2010). This can have devastating consequences on quality of life and increase the risk of suicidal thoughts and behaviors in the child population with ASD and epilepsy (Hofvander et al., 2009). Furthermore, it is important to take into account the existence of a possible sex bias when diagnosing depression in ASD and epilepsy, since females usually have a higher prevalence of depressive episodes than males. According to Vania and De La Barra (2013), this may be due to a later diagnosis, inadequate approaches due to lack of detection, and their own awareness of social difficulties, highlighting the need for improved diagnosis of emotional problems in people with ASD and epilepsy.

Difficulties in regulating emotions seem to characterize both ASD and epilepsy. Individuals with ASD and epilepsy often struggle with regulating negative affect, which is characterized by the existence of aversive emotional states such as nervousness, fear, disgust, guilt, anger, emotional distress, and non-pleasurable engagement (León Rodríguez and Cárdenas, 2020). They also exhibit characteristic emotional responses and self-regulation difficulties, making it harder to calm down once they are upset, compared to typically developing children (Rodríguez-Fernández et al., 2013). Therefore, it is crucial to address emotional needs in individuals with these two disorders, as their own manifestations, alongside behavioral and mental health problems, contribute to significantly higher levels of stress and anxiety observed in families of children with these characteristics (Allik et al., 2006; Lecavalier et al., 2006; Manning et al., 2015).

There is growing awareness of the importance of recognizing emotional and behavioral conditions as part of assessment and diagnosis (Begeer et al., 2008). However, reported rates of behavioral or emotional problems vary considerably from one study to another (Cano-Villagrasa et al., 2023). For instance, rates of anxiety reported by parents in children with ASD and epilepsy range from 22 to 84% (Gadow et al., 2005; Pearson et al., 2006; Simonoff et al., 2008; Kanne et al., 2009; White and Roberson-Nay, 2009; Mattila et al., 2010; MacNeil and Mostofsky, 2012), and stress problems have been identified in 31 to 80% of this population (Couturier et al., 2005; Oyane and Bjorvatn, 2005; Allik et al., 2006; Dominick et al., 2007; Krakowiak et al., 2008; Souders et al., 2009). Leekam et al. (2007) found that adverse sensory reactions occur in at least 90% of individuals with ASD and epilepsy and highlighted that these problems can cause disruptive and aggressive behavior in individuals with low autonomy and language impairments, as well as a low tolerance for frustration and emotional dysregulation. Moreover, in this study, it was observed that sensory reactions contribute to the occurrence of high rates of anxiety, which is also corroborated by the study of Kern et al. (2007). Lastly, in a population-based cohort study in the United Kingdom, Simonoff et al. (2008) investigated behavioral and emotional disorders in a sample of 112 children aged 5–10 years with ASD and epilepsy. The authors identified that 70% met criteria for at least one emotional or behavioral disorder, and 41% had two or more disorders, most commonly social anxiety and hyperactivity. These widely disparate prevalences are observed due to the large individual differences of each person with ASD and epilepsy, as well as the methodology and variables related to the home and school environment as relevant aspects determining emotional and behavioral problems in this population (Espinosa et al., 2018).

In addition to the marked behavioral and emotional profile of individuals with ASD and epilepsy, executive functions (EF) appear to be an area that is frequently affected in this comorbidity. The behavioral and emotional profile of individuals with ASD and epilepsy is significantly related to their executive functioning (López et al., 2016). EFs include a series of neurocognitive processes that enable intentional and goal-directed behavior, such as designing plans, setting goals, selecting appropriate behaviors, and inhibiting incorrect ones, cognitive flexibility, sustained attention, effective self-monitoring, and task organization (Zelazo and Carlson, 2012; Delgado Mejía and Etchepareborda Simonini, 2013; Schoemaker et al., 2013). In this line, one of the essential processes of EF is inhibitory control, defined as the ability to deliberately inhibit or suppress a dominant response (Diamond, 2013). The importance of inhibitory control lies in the fact that it enables individuals to control inappropriate behaviors in some contexts and to perform adequate responses to meet complex demands and to live adaptively in changing environments, being essential in the prevention of behavioral problems (Goldstein et al., 2007). Another basic process of executive functions is working memory, which, together with cognitive flexibility, facilitates the solution of social conflicts (Ziermans et al., 2012). Damasio and Maurer (1978) were the first researchers to link executive dysfunction in individuals with these disorders, as they share some common manifestations with people who have localized damage in the prefrontal cortex.

Among the most relevant symptomatology of the population with ASD and epilepsy regarding their executive functions are: absence of empathy, presence of stereotyped behaviors, perseverations, routines and restricted interests, compulsive behaviors, inappropriate and sudden emotional reactions, and lack of originality and creativity. The degree of alteration of EF varies depending on the age and level of impairment of this population. Thus, Han and Chan (2017) find significantly poorer performance in low-intelligence ASD children compared to those without intellectual deficits in three dimensions: planning, inhibitory control, and cognitive flexibility. Related to this, Hagberg et al. (2015) state in their study that high competence in verbal intelligence is a predictor of higher performance in inhibition, executive memory, and attention. Van Eylen et al. (2015) also show the existence of a strong association between variables related to age and intelligence quotient with executive functioning: inhibition of automatic responses, cognitive flexibility, spatial working memory, planning, and information processing speed. Finally, Chen et al. (2016) report in their comparative study of children with these disorders of different ages that children aged between 8 and 13 perform worse than the group of children aged between 13 and 18 in the dimensions of planning and working memory.

The previous discussion highlights the need to study EF in this population in order to characterize their functioning and develop research lines based on emotional and behavioral alterations, as well as their relationship with executive functioning in individuals with ASD and epilepsy (Mulas et al., 2001). However, there are problems in interpreting research findings due to the use of different sampling frames, sample sizes, criteria, and classifications in a large number of studies (Cala Hernández et al., 2015; Oviedo et al., 2015; Orduña and Barrio, 2021), making it difficult to obtain a global interpretation of the data. Further studies are needed to identify the precise rates of coexisting conditions and relevant risk and protective factors for planning appropriate services and interventions, and providing family support to minimize frustration and deterioration in children with ASD and epilepsy (Saeteros, 2022). The study conducted by Operto et al. (2021) reveals a significant negative correlation between adaptive behavior and emotional/behavioral problems in children diagnosed with Autism Spectrum Disorder (ASD). This suggests that individuals with higher levels of adaptive functioning tend to experience fewer emotional and behavioral difficulties. Moreover, the study establishes a noteworthy association between parental stress levels and the adaptive behavior as well as emotional/behavioral problems of children with ASD. Parents of children with higher levels of adaptive behavior tend to experience reduced stress, while those whose children exhibit more emotional and behavioral problems face heightened stress levels. Another study, such as the one conducted by Pastorino et al. (2021), explores the impact of these disorders and epilepsy on social cognition, which involves the ability to comprehend and respond to social and emotional signals from others. The findings demonstrate an impairment in social cognition in both individuals with ASD and epilepsy. People with these conditions encounter challenges in interpreting facial expressions, understanding others’ emotions, and engaging in appropriate social interactions. Furthermore, the study emphasizes the significance of considering the relationship between neurodevelopmental disorders and epilepsy in terms of their influence on social cognition. It is suggested that epilepsy may contribute to the observed difficulties in social cognition among individuals with ASD, although further research is required to gain a deeper understanding of this association. These findings underscore the importance of addressing both adaptive behavior and emotional/behavioral problems in children with ASD to enhance their quality of life and alleviate parental stress. Additionally, they emphasize the necessity of providing adequate support and resources for parents who face challenges related to caring for children with ASD (Volk, 2019).

Therefore, the main objective of the present study is to compare emotional and behavioral problems in three groups: a group of children with ASD, a group of children with epilepsy, and a group of children with ASD and epilepsy. Thus, the first hypothesis proposed for this study is that internalizing problems (depression, anxiety, social anxiety, and somatic complaints) will be more prominent in the group of children diagnosed with ASD and epilepsy than in the ASD and epilepsy groups alone. Likewise, the second research hypothesis is that externalizing problems (attention problems, hyperactivity-impulsivity, anger control problems, aggression, and defiant behavior) will be more pronounced in the group of children with ASD and epilepsy than in the ASD and epilepsy groups alone. Finally, the third research hypothesis proposed for this study is that participants with ASD and epilepsy will have greater executive functioning impairments than those with ASD or epilepsy alone.

## Methods

### Participants

**Figure 1:**
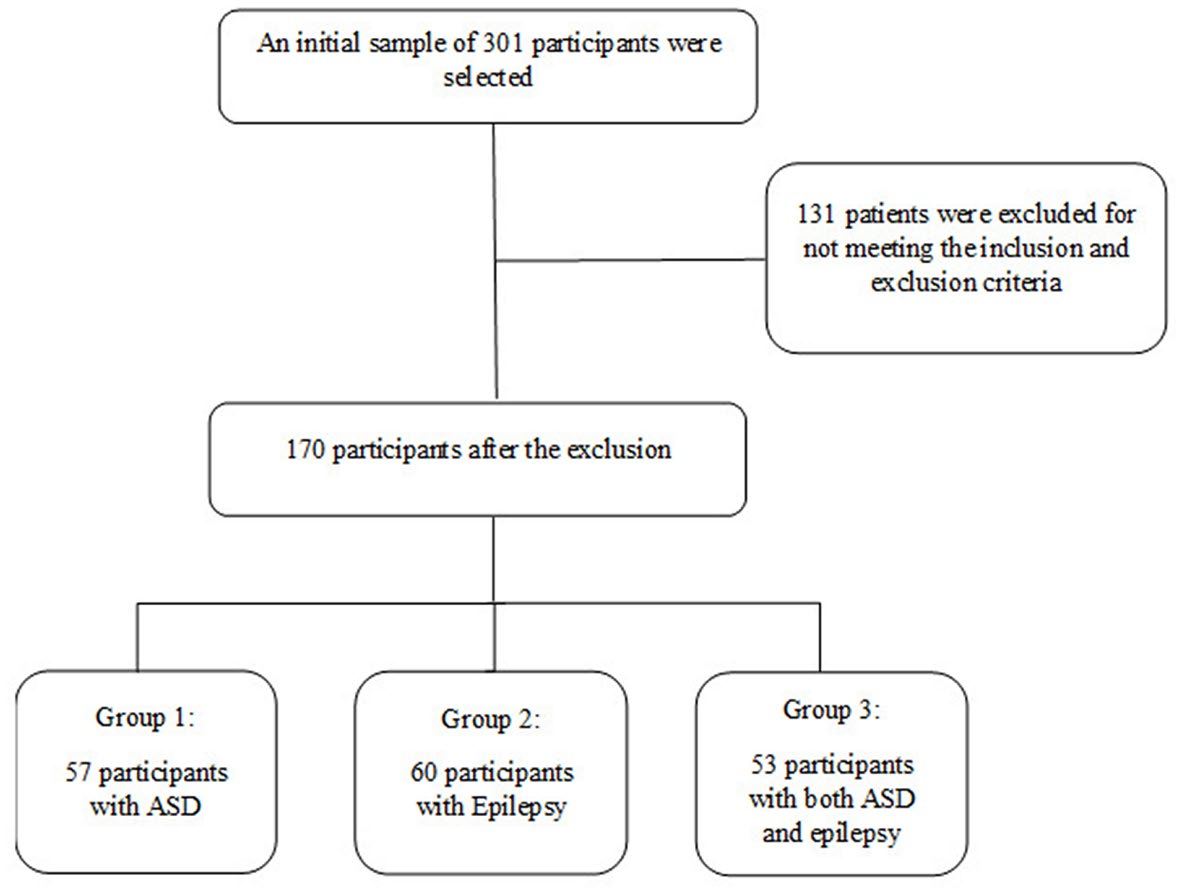
Participant selection procedure.

An initial sample of 301 subjects diagnosed with ASD, epilepsy, or both disorders was selected and underwent a rigorous screening process (Figure 1). The inclusion criteria were: being between 7 and 9 years old and having a diagnosis of ASD or epilepsy issued by a public health center. All participants diagnosed with ASD or epilepsy were evaluated at the Child and Adolescent Psychiatry and Pediatric Neurology Unit attached to their reference medical center by the multidisciplinary professional team of this unit. In these centers, a diagnostic screening evaluation was carried out through the administration of the M-CHAT-R questionnaire (Dereu, 2013). Those children who scored indicating a possible suspicion of alarm proceeded to use the ADOS-2 (Lord et al., 2008) and ADI-R (Rutter et al., 2006) protocols to confirm the diagnostic suspicion of ASD. Likewise, participants were evaluated by the neuropediatric team, conducting a neurological evaluation through Magnetic Resonance Imaging, Sleep-Deprived Electroencephalogram, and genetic tests that helped confirm both diagnoses. Finally, to control the variable of intellectual disability, all participants were administered the Wechsler Intelligence Scale for Children-IV (WISC-IV, Wechsler, 2010), excluding those with an IQ below 60.

After the initial selection of 301 subjects, a series of exclusion criteria were established, including having a motor or sensory disorder that would prevent study participation and having a moderate, severe, or profound intellectual disability. After the selection process, 131 subjects were excluded, resulting in a final sample of 170 participants between the ages of 7 and 9, divided into three groups: the ASD group (*n* = 57), the ASD and epilepsy group (*n* = 53), and the epilepsy group (*n* = 60). The ASD group (G1) consisted of 36 boys and 21 girls (*M*_age_ = 8.1; SD = 0.9) with a diagnosis of grade 3 ASD. The Epilepsy group (G2) consisted of 41 boys and 19 girls (*M*_age_ = 8.6; SD = 0.4). The ASD and epilepsy group (G3) consisted of 27 boys and 26 girls (*M*_age_ = 7.8; SD = 0.8), who also had a grade 3 ASD diagnosis and were evaluated at their reference medical center. This age range was chosen because assessment tests can be directly administered to study participants at age 6. Additionally, the diagnosis of ASD is established at age 6, and participants have had several years of speech and language therapy and neuropsychological intervention. Table 1 summarizes the main characteristics of the participants who made up the sample group of this study.

**Table 1:** Characteristics of the study participants.

Characterization of the participants			
		*N*	Percentage
Sex	Male	104	61.2
	Woman	66	38.2
Age	7 years old	47	27.6
	8 years old	61	35.9
	9 years old	62	36.5
Diagnosis	ASD	57	33.5
	ASD + Epilepsy	53	31.2
	Epilepsy	60	35.3
Years of treatment	2 years	34	20.0
	3 years	73	42.9
	4 years	63	37.1
Grade of incapacity	Less than 33%	52	29.1
	Between 33 and 66%	74	40
	More than 66%	44	30.9
Educational supports at school	Hearing and Language Teacher	41	30.6
	Teacher of Hearing and Language and Therapeutic Pedagogy	87	51.2
	Hearing and Language Teacher, Therapeutic Pedagogy and Educator	42	24.7
Gestation weeks	30–35	52	30.6
	35–40	87	51.2
	More than 40	31	18.2
Apgar	With risk	77	45.3
	Intermediate	63	37.1
	Normal	30	17.6
Intellectual disability	No presence	123	72.4
	Mind	47	27.6
Pharmacological therapy	No presence	52	30.6
	Valproate	41	24.1
	Risperidone	19	11.2
	Clonazepam	58	34.1

Finally, the following inclusion and exclusion criteria were established. On the one hand, the inclusion criteria were: (I) being between 5 and 8 years of age, (II) presenting a diagnosis of ASD or epilepsy issued by a public health agency or center, and (III) undergoing rehabilitation treatment at a rehabilitation clinic. On the other hand, the exclusion criteria were: (I) presenting a motor or sensory disease or disorder that hinders or prevents the correct performance of the study, (II) not presenting any communication or language at 5 years of age, and (III) presenting a moderate, severe or profound intellectual disability.

### Instruments

#### Evaluation system for children and adolescents

The SENA questionnaire, system for the evaluation of children and adolescents (SENA; Fernández et al., 2015) consists of a total of 9 questionnaires aimed at three age levels: Infant (3–6 years), Primary (6–12 years), and Secondary (12–18 years). It includes specific questionnaires to gather information from different informants regarding the main contexts in which the child operates (questionnaires for family and school), as well as three self-report models according to the age of the evaluated person that are applied from 6 years old. In each of them, the informants must assess the frequency of the described behavior using a five-point scale (Never or almost never to Always or almost always), except for the self-report for 6–8 years, which includes a three-option scale: Yes, No, and Sometimes.

### Behavior assessment system for children and adolescents-3

The Behavior Assessment System for Children and Adolescents-3rd Edition (BASC-3; González et al., 2004) is an instrument used for clinically evaluating the emotions and behaviors of children and adolescents, detecting maladaptive disorders in the family and school contexts. The BASC-3 consists of a self-report form for the patient and two questionnaires for parents (P) and teachers (T). For the purpose of this research, only the latter two will be taken into account.

### Neuropsychological evaluation of executive functions in children

Neuropsychological Evaluation of Executive Functions in Children (ENFEN; Portellano et al., 2009), is an instrument that allows to determine the level of maturity and cognitive performance in activities related to EF in children aged 6–12 years on an individual basis. The battery consists of four tests (Verbal Fluency, Trail Making, Ring Construction, and Resistance to Interference) that measure different components of EF. The results allow for a deeper diagnosis and guidance for neuropsychological intervention, both in healthy children and in those with developmental delay or cognitive or emotional alterations derived from brain damage or dysfunction.

### Procedure

For the present study, participant selection was carried out following the inclusion and exclusion criteria for the sample (see Table 1; Figure 1), subsequently grouping them into three experimental groups. Participants and their families were evaluated in two 1-h sessions. Data from the measurement instruments were stored in protected databases, which were later analyzed by the research group members, checking the fulfillment of the research hypotheses. Finally, this study was reviewed by the ethics committee of the Universidad Católica San Antonio de Murcia (UCAM), proposing its favorable verdict with the code: CE052206.

### Data analysis

The statistical analysis of this quasi-experimental study was carried out using different statistical designs. First, the Kolmogorov-Smirnoff test was used to examine the normality of the distribution of the dependent variables that make up the study. However, a graphical position point analysis has been conducted through a splot Q-Q study, whose results indicate that there is a homogeneous distribution among the groups comprising the study. Next, multivariate analysis of covariance (MANCOVA) was used to observe the relationship between variables related to externalizing and internalizing emotional and behavioral problems, conduct problems, and executive functions. The objective of using the MANCOVA statistical analysis is to control for the variable “intellectual disability” in the model of the present study. In this way, differences between the two groups that make up the sample were explored. Subsequently, analysis of covariance (ANCOVA) tests was carried out to observe the individual differences of each of the variables in the two groups. Finally, to control for Type I error, Holm-Bonferroni correction was performed (Holm, 1979).

## Results

### Difference in internalized problems in the groups

The MANCOVA conducted to evaluate differences in measures of internalizing problems among the ASD, Epilepsy, and ASD with Epilepsy groups revealed the presence of statistically significant differences (Wilks’ Lambda = 0.104, *F*(6,92) = 328.000, *p* < 0.001, η^2^_P_ = 0.677). As can be seen in Table 2, the variables in which significant differences were obtained were depression, anxiety, social anxiety, and somatic complaints. The results of the ANCOVAs related to internalizing problems are presented in Table 2.

**Table 2:** Differences between the measures of the groups: ASD (G1), Epilepsy (G2) and ASD with epilepsy (G3) in behavioral and emotional problems internalized in the SENA test.

Internalized problems	G1 (*n* = 57)		G2 (*n* = 60)		G3 (*n* = 53)		*F*_(6,92)_	η^2^_P_	Differences between groups
	*M*	SD	*M*	SD	*M*	SD			
Depression	13.38	4.41	6.18	2.22	23.74	3.64	304.182^*^	0.785	G2 < G1 < G3
Anxiety	13.86	4.33	5.73	2.29	22.18	3.82	245.391^*^	0.746	G2 < G1 < G3
Social Anxiety	11.42	4.11	6.55	2.10	22.82	4.19	288.063^*^	0.775	G2 < G1 < G3
Somatic complaints	13.74	4.27	6.30	2.16	23.26	3.87	287.669^*^	0.775	G2 < G1 < G3

* *p* < 0.05.

*M* (Mean); SD (Standard Deviation).

### Differences in externalized problems in the groups

The MANCOVA conducted to evaluate the differences in measures of externalized problems among the groups of ASD, Epilepsy, and ASD with Epilepsy revealed the presence of statistically significant differences (Wilks’ Lambda = 0.093, *F*(7,92) = 326.000, *p* < 0.001, η^2^_P_ = 0.694). As can be seen in Table 3, the variables in which significant differences were found were attention problems, hyperactivity-impulsivity, anger control problems, aggression, and defiant behavior. On the other hand, the results of the ANCOVAs related to externalized problems are presented in Table 3.

**Table 3:** Differences between the measures of the groups: ASD (G1), Epilepsy (G2) and ASD with epilepsy (G3) in the behavioral and emotional problems externalized in the SENA test.

Externalized problems	G1 (*n* = 57)		G2 (*n* = 60)		G3 (*n* = 53)		F_(6,92)_	η^2^_P_	Differences between groups
	*M*	SD	*M*	SD	*M*	SD			
Attention problems	11.78	3.43	6.08	2.23	24.48	2.88	237.076^*^	0.740	G2 < G1 < G3
Hyperactivity-impulsivity	13.70	4.44	6.03	2.40	24.04	2.74	227.424^*^	0.731	G2 < G1 < G3
Anger control problems	12.36	4.35	6.32	2.38	24.12	3.05	289.035^*^	0.776	G2 < G1 < G3
Aggression	14.90	4.41	6.22	2.17	24.44	2.83	286.645^*^	0.774	G2 < G1 < G3
Defiant behavior	11.86	4.21	6.37	2.13	22.32	2.91	265.126^*^	0.760	G2 < G1 < G3

* *p* < 0.05.

M (Mean); SD (Standard Deviation).

### Differences in the behavioral profile in the groups

Finally, the MANCOVA performed to evaluate differences in behavioral profile measures among the groups with ASD, Epilepsy, and ASD with Epilepsy revealed the presence of statistically significant differences (Wilks’ Lambda = 0.025, F(7,92) = 300.000, *p* < 0.001, η^2^_P_ = 0.840). As can be seen in Table 4, significant differences were obtained in the variables that make up the questionnaire. On the other hand, the results of the ANCOVAs related to behavioral problems are presented in the following Table 4.

**Table 4:** Differences between the measures of the groups: ASD (G1), Epilepsy (G2) and ASD with epilepsy (G3) in the behavioral profile recorded by parents and teachers in the BASC-3.

Behavioral profile	G1 (*n* = 57)		G2 (*n* = 60)		G3 (*n* = 53)		F_(6,92)_	η^2^_P_	Differences between groups
	*M*	SD	*M*	SD	*M*	SD			
Composite, clinical, and adaptive scale scores									
Aggressiveness	23.18	3.70	28.72	2.91	14.00	4.29	280.136^*^	0.770	G3 < G2 < G1
Anxiety	22.76	3.78	28.97	3.37	12.76	4.63	187.522^*^	0.692	G3 < G2 < G1
Depression	22.06	3.37	28.32	3.44	11.02	2.99	203.063^*^	0.709	G3 < G2 < G1
Somatization	21.78	3.74	28.03	2.99	11.62	3.56	247.547^*^	0.748	G3 < G2 < G1
Atypicality	20.80	3.48	28.53	2.87	11.14	3.43	200.885^*^	0.706	G3 < G2 < G1
Retreat	20.98	3.60	27.83	3.28	11.12	3.07	217.828^*^	0.723	G3 < G2 < G1
Attention problems	22.44	3.28	28.12	3.13	11.12	2.88	310.423^*^	0.788	G3 < G2 < G1
Adaptability	21.24	3.05	27.78	3.17	11.16	3.25	256.461^*^	0.754	G3 < G2 < G1
Social skills	21.84	3.65	28.00	3.10	11.06	2.69	284.131^*^	0.773	G3 < G2 < G1
Daily activities	21.06	3.38	28.40	3.44	11.24	3.31	203.021^*^	0.709	G3 < G2 < G1
Functional communication	21.86	2.98	28.27	3.08	11.08	3.06	296.367^*^	0.780	G3 < G2 < G1
Content scale scores									
Anger control	22.32	3.15	28.13	3.10	10.38	2.98	323.198^*^	0.795	G3 < G2 < G1
Bullying	22.04	3.38	27.97	3.00	9.90	3.05	304.054^*^	0.785	G3 < G2 < G1
Social development disorders	21.42	3.62	27.72	3.21	11.52	3.33	236.167^*^	0.739	G3 < G2 < G1
Emotional self-control	21.32	3.64	27.58	2.90	11.04	2.55	278.836^*^	0.770	G3 < G2 < G1
Executive functioning	21.32	3.82	27.87	3.18	10.08	3.02	247.922^*^	0.748	G3 < G2 < G1
Negative emotionality	21.28	3.43	27.83	2.96	10.46	3.45	248.719^*^	0.749	G3 < G2 < G1
Resilience	22.04	3.31	28.42	3.06	11.12	3.28	264.604^*^	0.760	G3 < G2 < G1

* *p* < 0.05.

M (Mean); SD (Standard Deviation).

### Differences in executive functions in the groups

The MANCOVA performed to evaluate the differences in executive functioning measures among the groups of ASD, Epilepsy, and ASD with Epilepsy revealed the presence of statistically significant differences (Wilks’ Lambda = 0.126, *F*(4,95) = 328.000, *p* < 0.001, η^2^_P_ = 0.645). As can be seen in Table 5, the variables in which significant differences were obtained were verbal fluency, trail making test, block design, and interference control. On the other hand, the results of the ANCOVAs related to executive functioning are presented in Table 5.

**Table 5:** Differences between the measures of the groups: ASD (G1), and Epilepsy (G2) and ASD with epilepsy (G3) in the measures of executive functioning in the ENFEN.

Executive functioning	G1 (*n* = 57)		G2 (*n* = 53)		G3 (*n* = 60)		F _(6,92)_	η^2^_P_	Differences between groups
	*M*	SD	*M*	SD	*M*	SD			
Verbal fluency	4.90	1.17	5.40	1.16	2.38	1.48	87.33^*^	0.511	G3 < G1 < G2
Trail Making test	25.78	3.17	24.83	8.17	36.45	6.91	56.20^*^	0.402	G1 < G2 < G3
Block design	268.97	84.83	123.58	67.40	303.32	24.27	119.11^*^	0.588	G2 < G1 < G3
Interference control	39.34	4.48	27.92	3.89	48.76	5.01	122.50^*^	0.595	G2 < G1 < G3

* *p* < 0.05.

M (Mean); SD (Standard Deviation).

## Discussion

It has been found that children with a comorbidity of ASD and epilepsy often experience emotional, behavioral, and executive functioning problems (Morgan and Lilienfeld, 2000; Riccio et al., 2011; Schoemaker et al., 2013; López Rodríguez and Cañadas Pérez, 2018; Ruggieri, 2020). However, discrepancies in the prevalence and methodologies of previous studies create a need for further research to better characterize this comorbidity.

Therefore, the main objective of this study was to compare emotional, behavioral, and executive functioning problems in a group of children with ASD, another group of children with ASD who also had epilepsy, and a last group that only had epilepsy. The first research hypothesis was that internalizing problems would be more pronounced in the group of children with ASD and epilepsy compared to those with only ASD and those with only epilepsy. The second research hypothesis was that externalizing problems would be more pronounced in the group of children with ASD and epilepsy compared to those with only ASD and those with only epilepsy. Finally, the third hypothesis was that the group of participants with ASD and epilepsy would present greater executive functioning impairments than those with only ASD and those with only epilepsy.

Overall, the results of this study indicate significant differences between the evaluated participant groups, especially that the group with ASD and the group with epilepsy show lower occurrence of internalizing and externalizing problems compared to the group of participants with comorbid ASD and epilepsy. This is related to the fact that individuals with ASD who do not have epilepsy show lower levels of anxiety or depression and fewer somatic complaints. Additionally, these participants exhibit fewer behavioral and attentional problems, which positively impacts their ability to perform basic activities of daily living and their inclusion in various contexts, compared to children with a diagnosis of comorbid ASD and epilepsy.

Regarding internalizing and externalizing emotional and behavioral problems, the results indicate that the group with ASD and the group with epilepsy obtain lower scores compared to the group with comorbid ASD and epilepsy. When the condition of comorbid ASD and epilepsy is present in the pediatric population, there is an increase in emotional and depressive disorders in these individuals due to genetic, environmental, and etiological factors of the disorder (Maskey et al., 2013). These findings are consistent with studies such as Tseng et al. (2011), which concludes that children with comorbid ASD and epilepsy are more likely to develop emotional and behavioral disorders than other neurodevelopmental disorders. The clinical profile described in that research indicates that individuals with comorbid ASD and epilepsy exhibit higher levels of anxiety and depression and have difficulties in socialization due to social anxiety problems. These findings are consistent with studies such as Canitano (2007), which provides a detailed description of emotional characteristics in children with comorbid ASD and epilepsy, showing how these children manifest significant alterations in emotional regulation, low frustration tolerance, and difficulties in effectively resolving social conflicts.

The present study is in line with the findings of previous scientific literature, as previous studies (Nordin and Gillberg, 1996; Canitano et al., 2005; Tuchman et al., 2010) indicate the general presence of emotional disturbances related to anxiety and depression in children with either ASD, epilepsy, or ASD with epilepsy, but do not compare these conditions in detail. The results of this study indicate that individuals with epilepsy show fewer alterations in the dimensions of depression, anxiety, social anxiety, and somatic complaints, compared to the other two groups (ASD and ASD with epilepsy), whose difficulties are more pronounced. This explains why ASD is a condition that significantly worsens these behavioral and emotional problems, being the determining disorder in the limitation of the anxious and depressive state of this pediatric population. Therefore, the internalized problems related to anxiety and depression in the population with ASD, who also have epilepsy, significantly limit the maturation development, as well as the adaptation to the environment of children with this clinical profile.

Secondly, regarding externalized emotional and behavioral problems, the results of our study reflect that the group with ASD and epilepsy presents higher scores than those presented in the ASD and epilepsy groups. Individuals with ASD and epilepsy show greater difficulties in attention processes, hyperactive or impulsive behavior, difficulty controlling anger, much more pronounced aggressive episodes, and defiant behavior than in the pediatric population that only presents ASD or epilepsy (Tuchman et al., 2005). The behavioral profile of children who only have ASD or epilepsy corresponds to high limitations in cognitive flexibility, emotional self-regulation (Mira et al., 2019), impulse control (Tárraga-Mínguez et al., 2021), or even the appearance of aggressive behaviors (Serna and Gallegos, 2021), although in those profiles that only present epilepsy, a slight improvement in these symptoms is observed (Muñoz, 2004).

These data suggest that the population with ASD and epilepsy is more limited in their emotional control and tends to exhibit more disruptive behaviors, therefore requiring special considerations in their care. This assertion is supported by studies such as those by del Barrio and Carrasco (2016), Juillerat et al. (2015), and Mena-Chamorro and Caqueo-Urízar (2020), which analyzed the emotional and behavioral problems exhibited by children with ASD or ASD with epilepsy. The results of these studies showed that there is greater emotional and behavioral disturbance exhibited in children with both ASD and epilepsy, as compared to those who do not present this comorbidity.

In ASD, the problem with emotional control and the emergence of social anxiety stems from difficulties in demonstrating empathy or recognizing the emotions of others, which generates difficulties in social relationships and affects the quality of life for both the child and their parents or significant figures. This can also be observed in the first years of life, as it is evident that the child does not present any type of attachment to their parents (Maseda Prats, 2013). Furthermore, in these children, emotional and behavioral control, which is responsible for modulating adaptation to different contexts, is affected because they generally do not use emotional or adaptive strategies, which means that reactions tend to be impulsive, irritating, and composed of aggressions, tantrums, or self-injuries, which in turn can be considered challenging. Additionally, empathy is affected, as it is one of the components that allows a person to understand both their own emotions and those of others (Mazefsky, 2015). Therefore, these problems will be magnified in our sample because ASD converges with epilepsy, damaging the main structures of emotion regulation, which implies a greater occurrence of anxiety and depression in these clinical profiles.

Finally, regarding executive functioning, the results of this study reveal that the group with ASD and epilepsy presents greater alterations than those shown by the ASD group and the Epilepsy group. These data can be interpreted to mean that individuals with ASD who also have epilepsy have greater difficulties in accessing functional lexicon, as well as in following an activity with high levels of sustained, selective, and divided attention, with task switching. This is in line with studies by Artigas (1999), Aparicio and Aguirre-García (2011), and García-Peñas (2009), which describe the behavioral and emotional characteristics of individuals with ASD and epilepsy. These studies indicate that children with this comorbidity show severe alterations in emotional management, as well as difficulties in behavior, significantly affecting skills such as planning and cognitive flexibility. Additionally, children with ASD and epilepsy show alterations in executive functioning, which implies difficulties in emotional and behavioral self-regulation, language, task switching, processing speed, and planning. All of this is consistent with studies by Pisón et al. (2022) and Mulas et al. (2001), which explored executive functions in various neurodevelopmental disorders, including ASD, and describe significant alterations in planning, inhibition, and emotional control, which are further compromised by the coexistence of epilepsy in these clinical profiles. These alterations negatively impact proper emotional management, as well as the application of self-regulation strategies that lead individuals with this disorder to develop aggressive or violent behaviors to resolve conflict situations, in addition to displaying impulsive behavior in environments such as schools and homes.

In summary, the population with ASD and epilepsy presents greater internalized emotional and behavioral problems related to depression, anxiety, social anxiety, and somatic complaints, as well as greater externalized emotional and behavioral problems related to attentional problems, hyperactivity or impulsivity, problems in anger control, aggression, and defiant behavior, compared to children who only have ASD or epilepsy. These disorders are explained by alterations in emotional and behavioral competencies, which compromise overall functioning of the individual.

However, this study has limitations regarding the selection of objective instruments rather than self-reported measures. The SENA questionnaire (Fernández et al., 2015) was administered to evaluate emotional and behavioral problems, which is filled out by family members based on their observations and experiences in different contexts where the child interacts. Although this is a test with a high ecological validity, other tests could be included that are directly administered by examiners to the participants. Despite these limitations, this study opens a line of work for future research and to respond to the needs of children with this profile and their families, who are also one of the most affected by the emotional and behavioral alterations characteristic of TEA, which are further increased by the co-occurrence of epilepsy. Lastly, social skills and the development of these competencies in different types of ASD could be included for future research and compared with other comorbid disorders.

In conclusion, the comorbidity between ASD and epilepsy has a high prevalence that has been increasing in recent years. The diagnosis of children with either of these disorders is becoming more common, which implies the need to determine and observe different factors and characteristics early and concisely to allow for early intervention in the emotional and behavioral alterations of the individual with this condition. The comorbid appearance of ASD and epilepsy implies a deficit in the proper developmental process of the child, seriously compromising emotional management and behavioral self-regulation. These limitations will significantly impair the proper performance of daily life activities, reducing the effectiveness and autonomy of the population with this comorbidity.

## Data availability statement

The raw data supporting the conclusions of this article will be made available by the authors, without undue reservation.

## Ethics statement

The studies involving human participants were reviewed and approved by Universidad Católica San Antonio de Murcia. Written informed consent to participate in this study was provided by the participants’ legal guardian/next of kin.

## Author contributions

AC-V designed the study, analyzed the data and prepared the draft. All the authors reviewed the writing of the manuscript and approved the version finally submitted.

## Conflict of interest

The authors declare that the research was conducted in the absence of any commercial or financial relationships that could be construed as a potential conflict of interest.

## Publisher’s note

All claims expressed in this article are solely those of the authors and do not necessarily represent those of their affiliated organizations, or those of the publisher, the editors and the reviewers. Any product that may be evaluated in this article, or claim that may be made by its manufacturer, is not guaranteed or endorsed by the publisher.
